# Ameliorative Potential of (-) Pseudosemiglabrin in Mice with Pilocarpine-Induced Epilepsy: Antioxidant, Anti-Inflammatory, Anti-Apoptotic, and Neurotransmission Modulation

**DOI:** 10.3390/ijms241310773

**Published:** 2023-06-28

**Authors:** Mohamed F. Balaha, Ahmed A. Alamer, Maged S. Abdel-Kader, Khalid M. Alharthy

**Affiliations:** 1 Clinical Pharmacy Department, College of Pharmacy, Prince Sattam Bin Abdulaziz University, Al-Kharj 11942, Saudi Arabia; 2 Department of Pharmacognosy, College of Pharmacy, Prince Sattam Bin Abdulaziz University, Al-Kharj 11942, Saudi Arabia; 3 Department of Pharmacology and Toxicology, College of Pharmacy, Prince Sattam Bin Abdulaziz University, Al-Kharj 11942, Saudi Arabia

**Keywords:** (-) pseudosemiglabrin, epilepsy, GABAergic and glutamatergic neurotransmission, Nrf2/HO-1 pathway, PI3K/Akt pathway, TLR-4/NF-κB pathway

## Abstract

One prevalent neurological disorder is epilepsy. Modulating GABAergic/glutamatergic neurotransmission, Nrf2/HO-1, PI3K/Akt, and TLR-4/NF-B pathways might be a therapeutic strategy for epilepsy. Eight-week-old BALB/c mice were administered 12.5, 25, or 50 mg/kg (-) pseudosemiglabrin orally one hour before inducing epilepsy with an i.p. injection of 360 mg/kg pilocarpine. (-) Pseudosemiglabrin dose-dependently alleviated pilocarpine-induced epilepsy, as revealed by the complete repression of pilocarpine-induced convulsions and 100% survival rate in mice. Furthermore, (-) pseudosemiglabrin significantly enhanced mice’s locomotor activities, brain GABA, SLC1A2, GABARα1 levels, glutamate decarboxylase activity, and SLC1A2 and GABARα1mRNA expression while decreasing brain glutamate, SLC6A1, GRIN1 levels, GABA transaminase activity, and SLC6A1 and GRIN1 mRNA expression. These potentials can be due to the suppression of the TLR-4/NF-κB and the enhancement of the Nrf2/HO-1 and PI3K/Akt pathways, as demonstrated by the reduction in TLR-4, NF-κB, IL-1β, TNF-α mRNA expression, MDA, NO, caspase-3, Bax levels, and Bax/Bcl-2 ratio, and the enhancement of Nrf2, HO-1, PI3K, Akt mRNA expression, GSH, Bcl-2 levels, and SOD activity. Additionally, (-) pseudosemiglabrin abrogated the pilocarpine-induced histopathological changes. Interestingly, the (-) pseudosemiglabrin intervention showed a comparable effect to the standard medication, diazepam. Therefore, (-) pseudosemiglabrin can be a promising medication for the management of epilepsy.

## 1. Introduction

Epilepsy is one of the world’s most common neurological disorders, characterized by recurrent seizures caused by sudden abnormal neuronal discharges in the brain that momentarily impair brain function. It affects more than 50 million people worldwide, kills 125,000 people each year, and is the sixth leading cause of disability worldwide; more than 85% of cases occur in low-income populations. As a result, it has a detrimental influence on health outcomes while also increasing healthcare expenses and the psychological, cognitive, social, and economic burden [1,2].

The search for new anti-epileptic drugs has concentrated chiefly on medications that suppress neuronal activity rather than cure the underlying illness [3]. Recently, astrocytes and microglia have received more attention because they play a crucial role in brain physiology. Aberrant astrocytes are typical hallmarks of epileptic foci in the human brain and experimental epilepsy models [4]. Normally, astrocyte glutamate transporters uptake some of the glutamic acid generated by the presynaptic membrane. Epilepsy has been associated with reduced astrocyte glutamate transporter expression, inhibited astrocyte glutamic acid uptake, and prompted astrocytes to release glutamate, thus altering brain homeostasis and the balance of excitatory and inhibitory neurotransmitters [5]. Furthermore, astrocytes produce several pro-inflammatory cytokines, including interleukin (IL)-1β and tumor necrosis factor-α (TNF-α), through the activation of the toll-like receptor 4/nuclear factor kappa-light-chain-enhancer of activated B cells (TLR-4/NF-κB) cascade, which can aggravate astrogliosis and augment epileptogenic inflammatory signaling [6,7]. Moreover, these pro-inflammatory cytokines accumulate rapidly in this vicious loop and influence synaptic transmission, altering neuronal excitability and leading to seizure genesis, oxidative stress, neuronal apoptosis, and damage, with additional worsening of the condition [8,9].

Additionally, recurring seizures are associated with decreased γ-aminobutyric acid (GABA) levels, increased GABA-transaminase activity, and decreased L-glutamate decarboxylase activity in cortical and hippocampus tissue, indicating that GABAergic neurotransmission is disturbed during epilepsy [9,10]. Furthermore, repeated seizures cause an increase in reactive oxygen and nitrogen species (ROS and RNS, respectively) via excitotoxicity, which changes protein function, membrane permeability, gene expression, and neuronal apoptosis [11]. These molecular dysfunctions have been linked to increased neuronal excitability, augmented pro-inflammatory cytokine levels, immune system exacerbation, and a reduction in the seizure threshold in an epilepsy patient [8,9,12].

The most common epilepsy, attributable to approximately 60% of all epileptic patients, is temporal lobe epilepsy, with nearly 30% of patients refractory to conventional pharmacological therapy [1,13]. Additionally, none of the anticonvulsants currently on the market work to resolve co-morbidities or the underlying pathophysiology of epilepsy; they merely treat the symptoms of epilepsy [14,15,16]. Furthermore, they have a lot of side effects, such as congenital anomalies, sedation, hepatotoxicity, agranulocytosis, colitis, drowsiness, and dizziness, and patients with epilepsy are more vulnerable to the side effects of anti-epileptic drugs, which are assumed to be due to structural or functional abnormalities [1,17,18]. Thus, more innovative disease-modifying medicines with really curative effects on epilepsy and safety are required while avoiding substantial side effects, particularly sedation, and medicinal plants are a source for producing more effective medications [12,19].

The major potential bioactive flavanone identified in Tephrosia species’ aerial portions is (-) pseudosemiglabrin [20,21,22]. It was recently discovered to have pivotal potentialities, including anti-inflammatory, neuroprotective, analgesic, antioxidant, antiplatelet, antidyslipidemic, diuretic, anti-cancer, and anti-angiogenic properties [20,21,22,23,24,25]. Additionally, Tephrosia purpurea extract, whose primary bioactive component is (-) pseudosemiglabrin, demonstrated potent anticonvulsant efficacy via antioxidant, anti-inflammatory, and GABAergic transmission modulation [26,27]. Therefore, we investigated the potential anti-epileptic properties of (-) pseudosemiglabrin in a mouse model of pilocarpine-induced epilepsy for its involvement in astrocytic and glial transporters, as well as GABAergic, glutamatergic, anti-inflammatory, antioxidant, and anti-apoptotic pathways, and the modulation of histopathological changes and neuronal loss.

## 2. Results

### 2.1. Compound Identification

**Figure 1:**
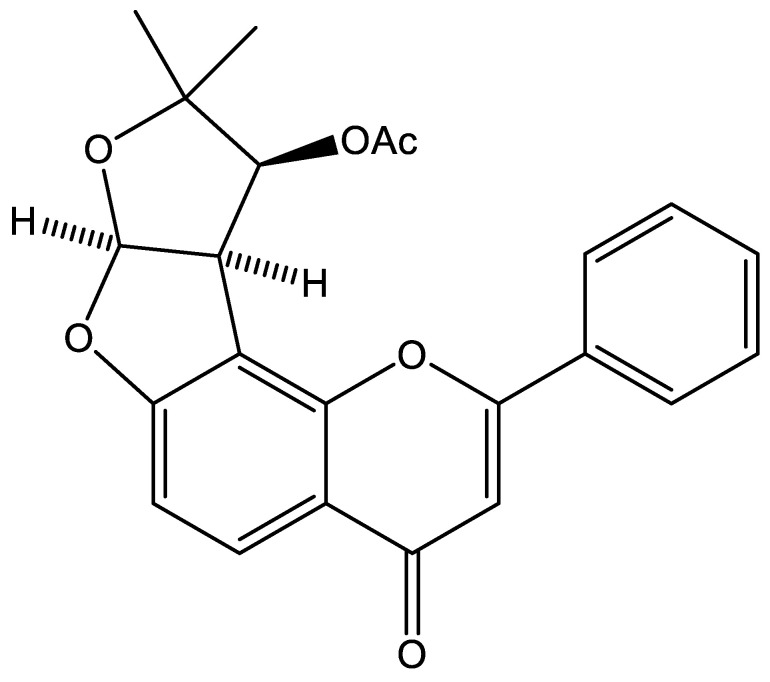
Chemical structure of (-) Pseudosemiglabrin.

(-) Pseudosemiglabrin: colorless crystals; m.p. 176.5 °C; [α]^25^_D_-404; UV λ_max_ MeOH: 258, 310 nm; ^1^H and ^13^C NMR identical with literature values [28]; HRESIMS [M+1]^+^ *m*/*z* 393.1331 (calcd. for C_23_H_20_O_6_+H, 393.1338) (Figure 1).

### 2.2. (-) Pseudosemiglabrin Mitigated Pilocarpine-Induced Convulsions and Mortality

Pilocarpine administration at a dose of 360 mg/kg produced convulsions and status epilepticus (SE) in all the experimental animals in the pilocarpine-induced convulsion (PIL) group, with a mortality rate of 70%. However, (-) pseudosemiglabrin treatment exhibited significant protection against pilocarpine-induced convulsion in a dose-dependent manner, as revealed by the prolongation of latency to first convulsion, reduction in convulsion, SE percentage, and seizures severity score; meanwhile, it increased the survival rate to 100% in the convulsion-induced group pretreated by pseudosemiglabrin 50 mg/kg (SSH group), compared with the PIL group. Moreover, both the SSH and convulsion-induced group pretreated by diazepam 5 mg/kg (DIZ) groups showed complete protection against pilocarpine-induced convulsion in all mice; however, the survival rate in the SSH group was 100%, but it was 95% in the DIZ group (Table 1).

### 2.3. (-) Pseudosemiglabrin Enhanced the Suppressed Mice’s Locomotor Activities Induced by Pilocarpine Injection

Pilocarpine injections significantly suppressed mice’s locomotor activities, as indicated by the reduction in mice’s crossing, rearing, and grooming numbers, with an increase in immobility number and latency to initiate locomotion compared with the control group. However, the pretreatment with (-) pseudosemiglabrin significantly enhanced the suppressed mice’s locomotor activities in a dose-dependent manner, as shown by the increase in mice’s crossing, rearing, and grooming numbers, as well as the reduction in immobility number and latency to initiate locomotion compared with the PIL group. In contrast, the pretreatment with diazepam significantly augmented the suppression of the mice’s locomotor activities induced by pilocarpine, as revealed by the further reduction in mice’s crossing, rearing, and grooming numbers, with the further increase in immobility number and latency to initiate locomotion compared with the PIL group (Table 2).

### 2.4. (-) Pseudosemiglabrin Modulated Mice’s Brain GABAergic and Glutamatergic Transmission

**Figure 2:**
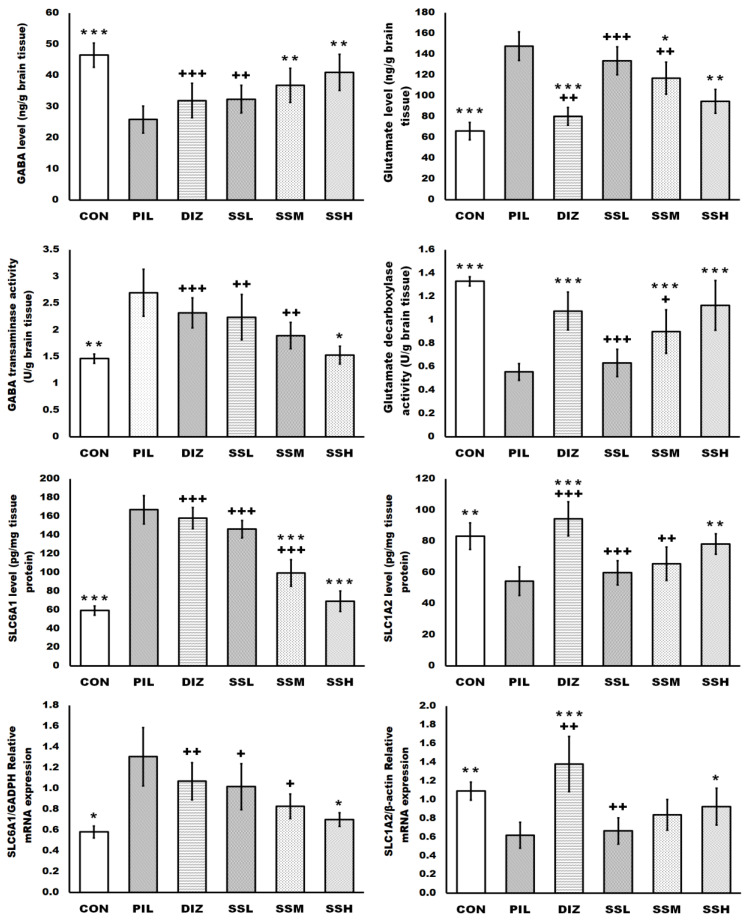
(-) Pseudosemiglabrin modulated mice’s brain GABAergic and glutamatergic transmission. The result was expressed as mean ± SD. CON: control group; PIL: pilocarpine-induced convulsion group; DIZ: convulsion-induced group pretreated by single oral dose of diazepam 5 mg/kg; SSL: convulsion-induced group pretreated by single oral dose of (-) pseudosemiglabrin 12.5 mg/kg; SSM: convulsion-induced group pretreated by single oral dose of (-) pseudosemiglabrin 25 mg/kg; SSH: convulsion-induced group pretreated by single oral dose of (-) pseudosemiglabrin 50 mg/kg. * *p* < 0.05, ** *p* < 0.01 and *** *p* < 0.001 (vs. PIL group), + *p* < 0.05, ++ *p* < 0.01 and +++ *p* < 0.001 (vs. SSH group).

(-) Pseudosemiglabrin dose-dependently and significantly enhanced the decreased brain GABA, solute carrier family 1 member 2 (SLC1A2) levels, glutamate decarboxylase activity, and SLC1A2 mRNA expression, as well as reduced the increased brain glutamate, sodium-and chloride-dependent GABA transporter 1 (SLC6A1) levels, GABA transaminase activity, and SLC6A1 mRNA expression induced by pilocarpine injection. Furthermore, the SSH group showed a more significant enhancement of brain GABA level and a more significant reduction in brain GABA transaminase activity, SLC6A1 level, and SLC6A1 mRNA expression compared with the DIZ group. However, the DIZ group exhibited a more significant reduction in the brain glutamate level and a more significant enhancement of the brain SLC1A2 level and mRNA expression than the SSH group. Nevertheless, there was no significant difference between the SSH and DIZ groups regarding the enhancement of the brain glutamate decarboxylase activity (Figure 2).

**Figure 3:**
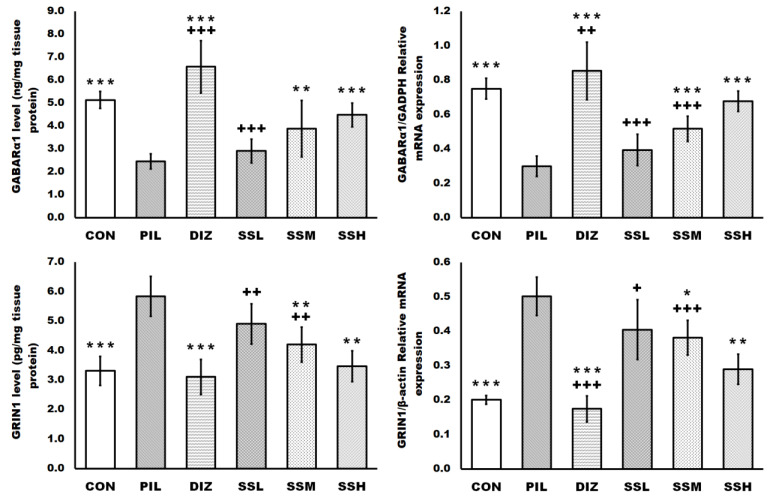
(-) Pseudosemiglabrin enhanced the brain GABARα1 and reduced the brain GRIN1 expression in a mouse model of pilocarpine-induced epilepsy. The result was expressed as mean ± SD. CON: control group; PIL: pilocarpine-induced convulsion group; DIZ: convulsion-induced group pretreated by single oral dose of diazepam 5 mg/kg; SSL: convulsion-induced group pretreated by single oral dose of (-) pseudosemiglabrin 12.5 mg/kg; SSM: convulsion-induced group pretreated by single oral dose of (-) pseudosemiglabrin 25 mg/kg; SSH: convulsion-induced group pretreated by single oral dose of (-) pseudosemiglabrin 50 mg/kg. * *p* < 0.05, ** *p* < 0.01 and *** *p* < 0.001 (vs. PIL group), + *p* < 0.05, ++ *p* < 0.01 and +++ *p* < 0.001 (vs. SSH group).

Parallel to the enhancement of the brain GABA level and the reduction in the brain glutamate level, (-) pseudosemiglabrin dose-dependently and significantly enhanced the reduced brain GABA receptor subunit alpha-1 (GABARα1) level and mRNA expression and reduced the increased glutamate ionotropic receptor NMDA type subunit 1 (GRIN1) level and mRNA expression, induced by pilocarpine. However, the DIZ group showed a more significant enhancement of the reduced brain GABARα1 level and mRNA expression and a more significant reduction in the increased GRIN1 mRNA expression than the SSH group. So far, there has been no significant difference between the DIZ and SHH groups regarding the reduction in the increased GRIN1 level (Figure 3).

### 2.5. (-) Pseudosemiglabrin Enhanced Mice’s Brain Antioxidant System Activities

**Figure 4:**
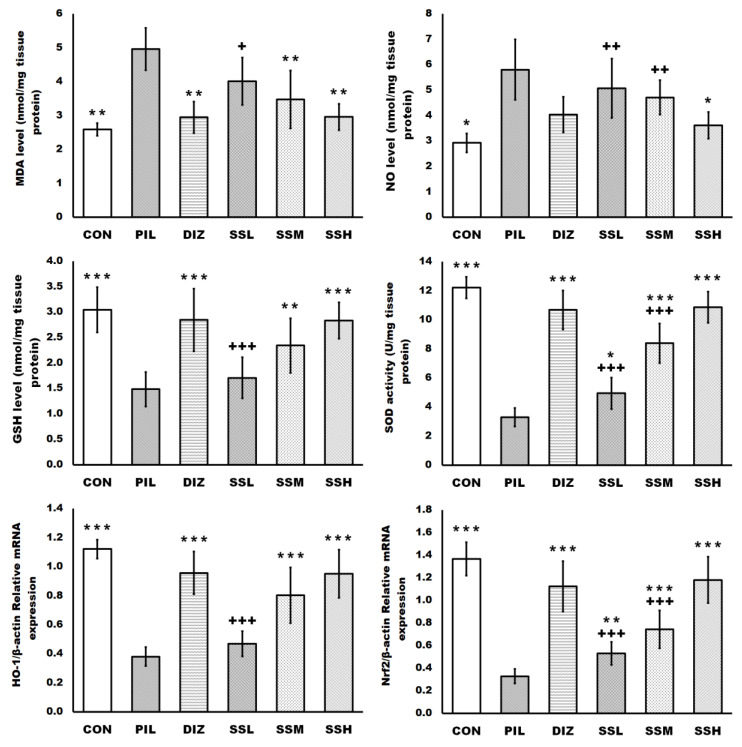
(-) Pseudosemiglabrin enhanced mice’s brain antioxidant system activities. The result was expressed as mean ± SD. CON: control group; PIL: pilocarpine-induced convulsion group; DIZ: convulsion-induced group pretreated by single oral dose of diazepam 5 mg/kg; SSL: convulsion-induced group pretreated by single oral dose of (-) pseudosemiglabrin 12.5 mg/kg; SSM: convulsion-induced group pretreated by single oral dose of (-) pseudosemiglabrin 25 mg/kg; SSH: convulsion-induced group pretreated by single oral dose of (-) pseudosemiglabrin 50 mg/kg. * *p* < 0.05, ** *p* < 0.01 and *** *p* < 0.001 (vs. PIL group), + *p* < 0.05, ++ *p* < 0.01 and +++ *p* < 0.001 (vs. SSH group).

The brain tissue antioxidant system is significantly disturbed by pilocarpine administration, as exhibited by the raised brain tissue malondialdehyde (MDA) and nitric oxide (NO) levels, and the reduction in brain tissue reduced glutathione (GSH) level, superoxide dismutase (SOD) activity, heme oxygenase-1 (HO-1), and nuclear factor erythroid 2-related factor 2 (Nrf-2) mRNA expression compared with the control group. Meanwhile, the (-) pseudosemiglabrin pretreatment significantly and dose-dependently restored the brain tissue antioxidant system, as evinced by the reduction in the raised brain tissue MDA and NO levels, and the enhancement of the reduced brain tissue GSH level, SOD activity, and HO-1 and Nrf-2 mRNA expression compared with the PIL group. Moreover, in contrast to the DIZ group, the (-) pseudosemiglabrin pretreatment in the SSH group reduced the elevated NO level induced by pilocarpine injection (Figure 4).

### 2.6. (-) Pseudosemiglabrin-Inhibited Neuronal Apoptosis Induced by Pilocarpine Injection

**Figure 5:**
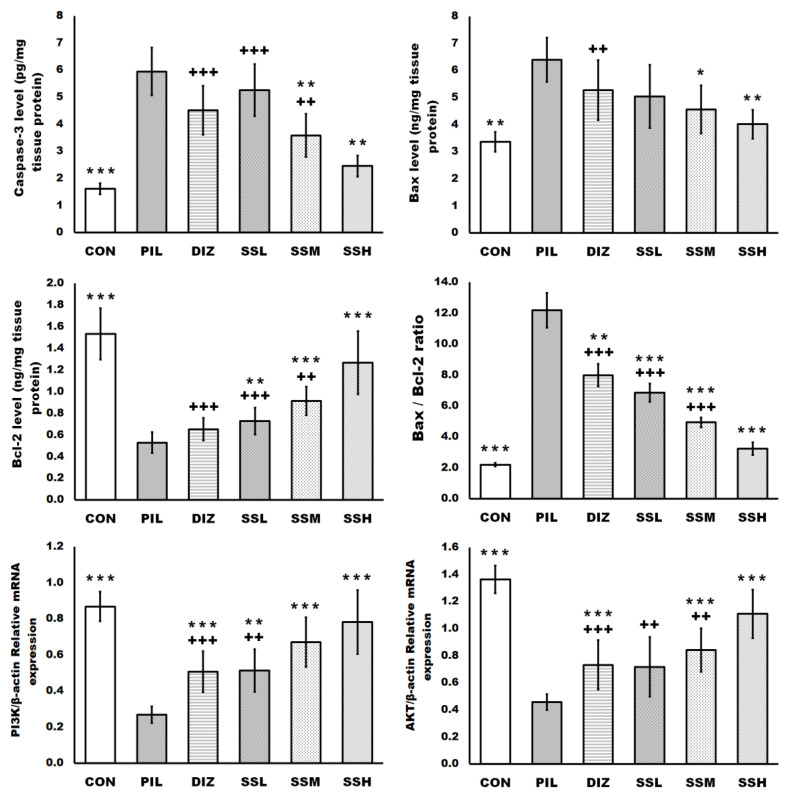
(-) Pseudosemiglabrin-inhibited neuronal apoptosis induced by pilocarpine injection. The result was expressed as mean ± SD. CON: control group; PIL: pilocarpine-induced convulsion group; DIZ: convulsion-induced group pretreated by single oral dose of diazepam 5 mg/kg; SSL: convulsion-induced group pretreated by single oral dose of (-) pseudosemiglabrin 12.5 mg/kg; SSM: convulsion-induced group pretreated by single oral dose of (-) pseudosemiglabrin 25 mg/kg; SSH: convulsion-induced group pretreated by single oral dose of (-) pseudosemiglabrin 50 mg/kg. * *p* < 0.05, ** *p* < 0.01 and *** *p* < 0.001 (vs. PIL group), ++ *p* < 0.01 and +++ *p* < 0.001 (vs. SSH group).

Pilocarpine injection enhanced neuronal apoptosis in the brain tissue, as shown by the increased caspase-3 and BCL2-Associated X Protein (Bax) levels, Bax/B-cell lymphoma two protein (Bcl-2) ratio, and the reduction in Bcl-2 level and the mRNA expression of phosphoinositide 3-kinase (PI3K) and protein kinase B (Akt) compared with the control group. Meanwhile, the pretreatment with (-) pseudosemiglabrin significantly inhibited the neuronal apoptosis induced by pilocarpine injection in a dose-dependent manner, as evidenced by the reduction in the increased caspase-3 and Bax levels and Bax/Bcl-2 ratio together with the promotion of the diminished Bcl-2 level and the mRNA expression of PI3K and Akt, compared with the PIL group. In contrast, the pretreatment with diazepam showed a non-significant effect on the increased caspase-3 and Bax levels and the decreased Bcl-2 level compared with the PIL group. Furthermore, the SSH group showed a more significant Bax/Bcl-2 ratio reduction and enhancement of PI3K and Akt mRNA expressions than the DIZ group (Figure 5).

### 2.7. (-) Pseudosemiglabrin Suppressed Brain Tissues’ Neuro-Inflammatory Signals Induced by Pilocarpine Injection

**Figure 6:**
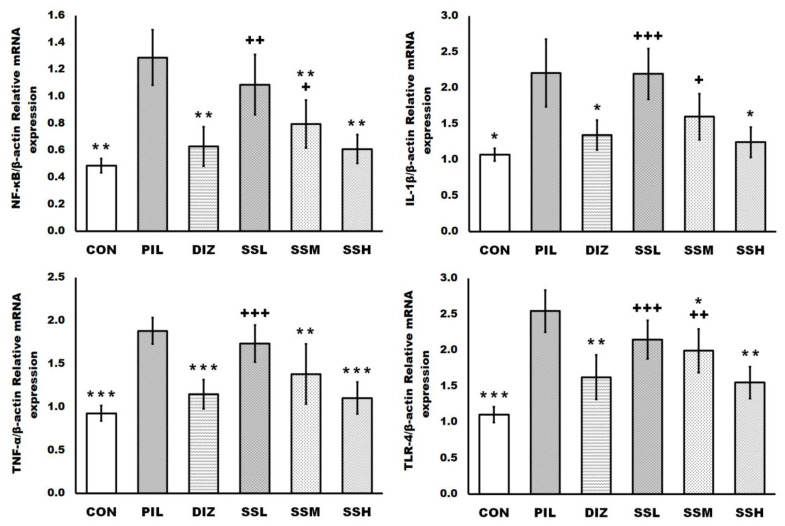
(-) Pseudosemiglabrin suppressed brain tissues’ neuro-inflammatory signals induced by pilocarpine injection. The result was expressed as mean ± SD. CON: control group; PIL: pilocarpine-induced convulsion group; DIZ: convulsion-induced group pretreated by single oral dose of diazepam 5 mg/kg; SSL: convulsion-induced group pretreated by single oral dose of (-) pseudosemiglabrin 12.5 mg/kg; SSM: convulsion-induced group pretreated by single oral dose of (-) pseudosemiglabrin 25 mg/kg; SSH: convulsion-induced group pretreated by single oral dose of (-) pseudosemiglabrin 50 mg/kg. * *p* < 0.05, ** *p* < 0.01 and *** *p* < 0.001 (vs. PIL group), + *p* < 0.05, ++ *p* < 0.01 and +++ *p* < 0.001 (vs. SSH group).

Pilocarpine administration activated the brain tissues’ neuro-inflammation, as revealed by the raised mRNA expression of TLR-4, NF-kB, IL-1β, and TNF-α compared with the control group. However, the pretreatment with (-) pseudosemiglabrin significantly and dose-dependently suppressed the brain tissues’ neuro-inflammatory signals, as it decreased the enhanced TLR-4, NF-kB, IL-1β, and TNF-α mRNA expression, compared with the PIL group. Moreover, there was no significant difference between the SSH and DIZ groups regarding the brain tissues’ neuro-inflammatory signal suppression (Figure 6).

### 2.8. (-) Pseudosemiglabrin Demotes the Brain Tissue’s Histopathologic Changes, Pathological Score, and Enhanced Neuronal Survival of the Hippocampal CA1 and CA3 Cells in Pilocarpine-Induced Convulsion

**Figure 7:**
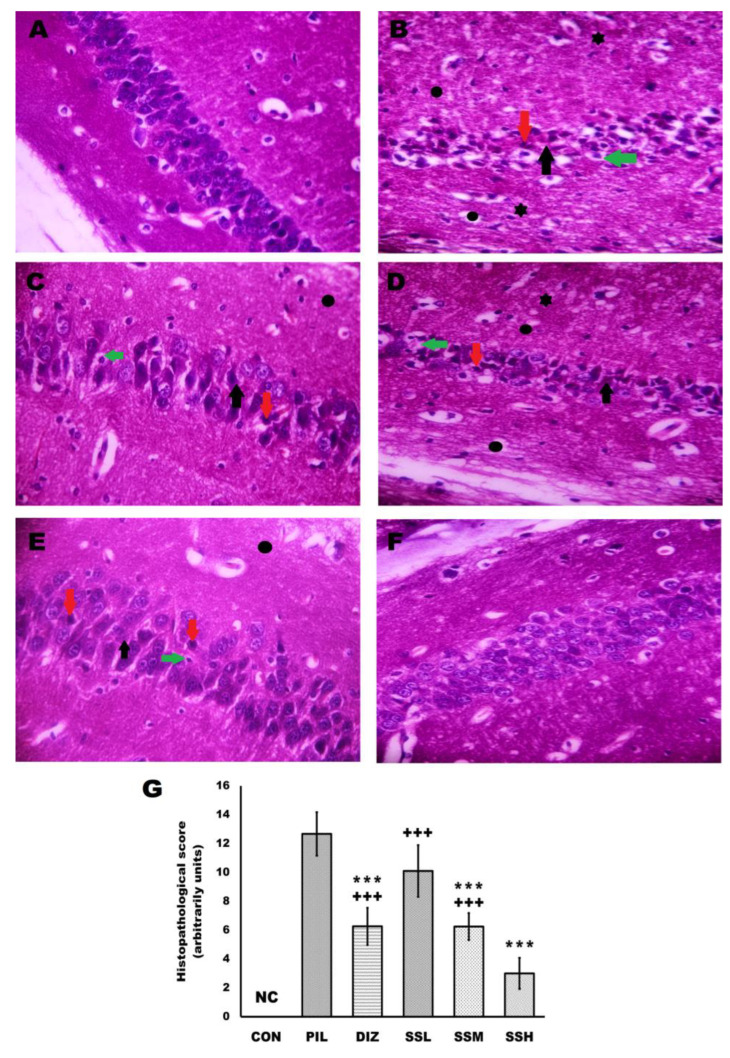
(-) Pseudosemiglabrin demotes the brain tissue’s histopathologic changes and histopathological score in pilocarpine-induced convulsion. (**A**) The control group displayed intact and neatly arranged neurons; (**B**) the pilocarpine-induced convulsion group displayed severe neuronal degeneration; (**C**) the convulsion-induced group pretreated by diazepam displayed a mild neuronal degeneration; (**D**–**F**) convulsion-induced group pretreated by (-) pseudosemiglabrin 12.5, 25, 50 mg/kg, respectively, displayed a dose-dependent mitigation of pilocarpine-induced neurodegeneration to be nearly like the control group in the SSH group. Red arrow: nuclear pyknosis; green arrow: cytoplasmic vacuolation; black arrow: neuronal necrosis; black circle: cerebral congestion; black asterix: inflammatory influx. (**G**) Histopathological score. NC: no change, *** *p* < 0.001 (vs. PIL group) and +++ *p* < 0.001 (vs. SSH group) (H and E, X400).

The hematoxylin and eosin (H and E)-stained sections of the control group displayed intact and neatly arranged neurons. However, in the PIL group, there was intense cell damage exhibited by nuclear pyknosis, cytoplasmic vacuolation, and neuronal necrosis, along with cerebral congestion and inflammatory influx throughout various brain areas compared with the control group. (-) Pseudosemiglabrin pretreatment noticeably mitigated the pilocarpine-induced neurodegeneration in a dose-dependent manner, to be nearly like the control group in the SSH group. Statistically, (-) pseudosemiglabrin pretreatment significantly and dose-dependently abrogated the histopathological changes induced by pilocarpine injection, which was revealed by the significantly reduced histopathological score. The SSH group also demonstrated more reduction in the elevated histopathological score than the DIZ group (Figure 7).

**Figure 8:**
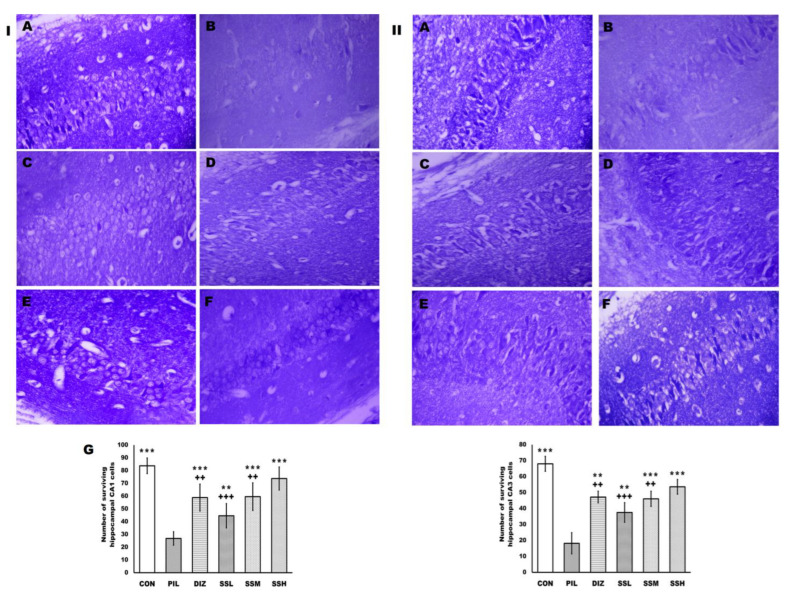
(-) Pseudosemiglabrin enhanced neuronal survival of the hippocampal CA1 and CA3 cells in pilocarpine-induced convulsion. (**I**) Nissl’s stain of CA1 hippocampal region; (**II**) Nissl’s stain of CA3 hippocampal region; (**A**) control group; (**B**) pilocarpine-induced convulsion group; (**C**) convulsion-induced group pretreated by diazepam; (**D**–**F**) convulsion-induced group pretreated by (-) pseudosemiglabrin 12.5, 25, and 50 mg/kg, respectively; (**G**) number of surviving hippocampal cells, ** *p* < 0.01 and *** *p* < 0.001 (vs. PIL group), ++ *p* < 0.01 and +++ *p* < 0.001 (vs. SSH group) (Nissl’s stain, X400).

Additionally, Nissl’s staining showed many pyramid-shaped neurons with dense cytoplasmic granules and Nissl’s bodies in the hippocampal CA1 and CA3 regions of the control group. However, pilocarpine injection induced a significant neuronal loss in the hippocampal CA1 and CA3 regions compared with the control group, and the remaining cells were irregular, swollen, and necrotic. Nevertheless, (-) pseudosemiglabrin pretreatment effectively ameliorated neuronal degeneration in the hippocampal CA1 and CA3 regions, as well as significantly and dose-dependently increasing the neuronal survival of the hippocampal CA1 and CA3 regions compared with the PIL group. Moreover, the SSH group demonstrated a more significant increase in the neuronal survival of the hippocampal CA1 and CA3 regions than the DIZ group (Figure 8).

## 3. Discussion

(-) Pseudosemiglabrin used in the present study was identified by comparing the spectroscopic data with those reported in the literature [28]. The current study’s data confirmed the dose-dependent significant anti-epileptic effectiveness of (-) pseudosemiglabrin in a mouse model of pilocarpine-induced epilepsy, as evidenced by the complete avoidance of pilocarpine-induced convulsions and the 100% survival rate of mice in the SSH group. Furthermore, (-) pseudosemiglabrin considerably increased the mice’s locomotor activities, reduced by pilocarpine treatment, as seen by an increase in crossings, rearings, and groomings, as well as a decrease in immobility and latency to initiating locomotion. Additionally, (-) pseudosemiglabrin significantly increased the decreased brain GABA, SLC1A2 levels, glutamate decarboxylase activity, and SLC1A2 mRNA expression induced by pilocarpine injection while decreasing the increased brain glutamate, SLC6A1 levels, GABA transaminase activity, and SLC6A1 mRNA expression. Moreover, (-) pseudosemiglabrin boosted the decreased brain GABARα1 level and mRNA expression while decreasing the elevated GRIN1 level and mRNA expression caused by pilocarpine injection. Moreover, (-) pseudosemiglabrin markedly restored the brain tissue antioxidant system, as evidenced by a decrease in elevated brain tissue MDA and NO levels and an increase in lowered brain tissue GSH level, SOD activity, and HO-1 and Nrf-2 mRNA expression induced by pilocarpine injection.

Furthermore, (-) pseudosemiglabrin-inhibited neuronal apoptosis induced by pilocarpine injection in a dose-dependent manner, as evidenced by the reduction in increased caspase-3 and Bax levels, the Bax/Bcl-2 ratio, and the promotion of decreased Bcl-2 level and PI3K and Akt mRNA expression. In addition, (-) pseudosemiglabrin significantly suppressed the neuro-inflammatory signals in the brain tissues, as it decreased the increased TLR-4, NF-kB, IL-1β, and TNF-α mRNA expression, as well it increased the number of surviving hippocampal neurons in the CA1 and CA3 regions, and reversed the H and E histopathological changes caused by pilocarpine injection. Interestingly, the (-) pseudosemiglabrin intervention showed a considerable neuroprotective effect on the treated mice, comparable to the traditional medication diazepam.

Curing refractory epilepsy requires the use of the appropriate epilepsy model. Acute animal models, such as the pilocarpine-induced epilepsy model, are believed to be useful for initial anti-epileptic pharmacological screening [29]. The pilocarpine epilepsy model resulted in persistent epileptic-like disorders that accurately mimic the symptoms and pharmacology of the most common type of human epilepsy, refractory temporal lobe epilepsy, and helped the studying of the pathophysiology, behavioral, and electroencephalographic aspects of epilepsy [1,30,31,32]. Pilocarpine is a nonselective muscarinic agonist with a relatively high affinity for muscarinic receptors in the central nervous system. When administered intraperitoneally, it induces tonic-clonic seizures since it activates excitatory neurons in specific brain regions, including the amygdala, hippocampus, and entorhinal cortex, with glutamate release [31,32,33,34].

Traditional anti-epileptic drugs frequently cause sedation and dizziness as adverse effects, and patients with epilepsy are more vulnerable to the side effects of anti-epileptic drugs, potentially due to structural or functional abnormalities [17]. In this study, the open-field test revealed that mice’s locomotor activity was suppressed, as evidenced by a decrease in the number of crossings, rearings, and groomings, as well as an increase in the immobility and latency to initiating locomotion in the PIL group, indicating anxiety-like behavior in mice [32]. In the current study, diazepam, a traditional anti-epileptic medicine that acts as a positive allosteric modulator of GABA_A_ receptors, causes sedation and dizziness by boosting GABA action, thus exacerbating the locomotor activity reduction produced by pilocarpine, which was thought to be related to sedation induction in mice [35,36]. However, (-) pseudosemiglabrin pretreatment enhanced mice’s locomotor activity, indicating anxiolytic-like effects in mice without sedation; this might be owing to interaction with GABAergic neurotransmission. The absence of inhibition of locomotor activity, an indicator of alertness, contributes to the potential safety of (-) pseudosemiglabrin for drug development.

The current study found that pilocarpine injection decreased brain GABA, SLC1A2, and GABARα1 levels, glutamate decarboxylase activity, and SLC1A2 and GABARα1 mRNA expression while increasing brain glutamate, SLC6A1, GRIN1 levels, GABA transaminase activity, and SLC6A1 and GRIN1 mRNA expression. As a result of these data, epilepsy can be induced by a disruption in GABAergic and glutamatergic neurotransmission. These findings are consistent with earlier findings [1,9,13,16,37,38].

Astrocytes are recognized to have a role in regulating neurotransmitter concentrations, such as GABA and glutamate, as well as neuronal homeostasis and immunological response [5]. The primary inhibitory neurotransmitter in the brain is GABA, and boosting GABA neurotransmission has been proven to reduce seizures [9]. Blocking the glutamate decarboxylase enzyme, which is responsible for GABA generation from glutamate, and the SLC1A2 transporter, which is responsible for glutamate uptake, or boosting the GABA transaminase enzyme, which is responsible for GABA metabolism, and the astrocytic SLC6A1 transporter, which is the crucial brain GABA transporter that is responsible for GABA uptake, cause epilepsy [1,16,35,39]. In the current study, (-) pseudosemiglabrin enhanced glutamate decarboxylase enzyme and SLC1A2 transporter while suppressing GABA transaminase enzyme and SLC6A1 transporter, thereby raising brain GABA and decreasing brain glutamate levels and supporting its anti-epileptic abilities. Moreover, (-) pseudosemiglabrin, in the current study, enhanced brain GABA level and suppressed GABA transaminase activity and SLC6A1 transporter more than diazepam. However, diazepam suppressed the glutamate level and enhanced the SLC1A2 transporter more than (-) pseudosemiglabrin, this may have contributed to the anti-epileptic effect of (-) pseudosemiglabrin without sedation induction, and thus it may be more beneficial for epileptic patients.

It is well-recognized that downregulation of the N-methyl-D-aspartate receptors (NMDAR) has neuroprotective properties, suppressing epilepsy development and protecting anti-epileptic treatment refractoriness [40]. Furthermore, activated astrocytes affect not only glutamic acid and GABA secretion but also regulate the expression of the neuronal NMDAR, suggesting that astrocytes may be necessary for modulating epileptogenesis [8]. Grin 1 mRNA expression is usually used to investigate NMDAR expression in epileptic mice’s hippocampus [8,37]. Additionally, GABARα1 is the most widely expressed GABAR subunit in brain neurons, with high expression levels in the temporal cortex and hippocampus, which is markedly downregulated in epilepsy [15]. Data from the present study revealed that pretreatment with (-) pseudosemiglabrin, upregulated GABARα1, and downregulated GRIN1 levels and mRNA expressions may have contributed to the anti-epileptic efficacy of (-) pseudosemiglabrin. However, (-) pseudosemiglabrin demonstrated a less substantial capacity to upregulate GABARα1 level and mRNA expression and downregulate GRIN1 mRNA expression than diazepam, which may be ascribed to (-) pseudosemiglabrin’s non-sedating, anti-epileptic efficiency.

The brain tissue is susceptible to free radical damage due to its high rate of oxidative metabolism and limited antioxidant defenses [41]. Excessive glutamate stimulation of postsynaptic NMDARs is believed to be involved in epileptic cell death. Glutamate hyperactivation of NMDARs promotes neuronal excitotoxicity and degeneration via many pathways, including nitric oxide synthase activation, ROS, and RNS production [37]. The pathophysiology of pilocarpine-induced epilepsy is considered to include both oxidative and nitrosative stress, as persistent seizures cause an increase in mitochondrial oxidative and nitrosative stress, with subsequent cell damage [13,29,37].

The PIL group in the current study exhibited a disruption of the brain antioxidant system, which was considerably alleviated by (-) pseudosemiglabrin pretreatment, with considerable mitigation of epilepsy-induced reductions in SOD activity and GSH level. Moreover, compared with the control group, pilocarpine injection significantly increased brain MDA and NO levels; however, (-) pseudosemiglabrin pretreatment resulted in a dose-dependent reduction in raised MDA and NO levels compared with the PIL group. These findings support prior research on the antioxidant potentiality of (-) pseudosemiglabrin and Tephrosia species, in which (-) pseudosemiglabrin is the predominant biologically active component [22,23,25,26,27]. Additionally, these effects are comparable to those of diazepam, a medicine known to decrease oxidative stress; nevertheless, (-) pseudosemiglabrin considerably lowered the elevated NO level, but diazepam did not [42,43].

Moreover, the activation of the Nrf2/HO-1 signaling pathway improves endogenous antioxidant defense and provides neuroprotection against pilocarpine-induced seizures [44,45,46]. The activated Nrf2/HO-1 pathway was detected in this study, as evidenced by enhanced Nrf-2 and HO-1 mRNA expression after (-) pseudosemiglabrin administration. Additionally, our results showed that (-) pseudosemiglabrin exhibited anti-epileptic effects in conjunction with Nrf2/HO-1 pathway activation, thereby reducing seizure-induced neuronal damage that may be partially attributed to (-) pseudosemiglabrin’s direct impact on the Nrf2/HO-1 pathway activation.

Additionally, activation of the PI3K/Akt pathway is essential for neuronal survival as it promotes cell survival while blocking apoptosis [29]. Akt, a serine/threonine kinase, is the primary mediator of PI3K-initiated signaling and can increase neuronal survival by modulating caspases, Bcl-2, and Bax expression [47]. It is well-established that oxidative stress may trigger neuronal apoptosis, and caspase family proteins are the cell death executors. Caspase-3 is an essential death protease used as a biomarker to determine if cells are experiencing apoptosis [38]. Furthermore, Bcl-2 has been identified as having anti-apoptotic, antioxidant, and cytoprotective activities [29]. On the other hand, Bax can directly stimulate cytochrome c production and release to the cytoplasm, trigger caspase-3 activation, and block anti-apoptotic Bcl-2 proteins [11]. Likewise, stimulation of the PI3K/Akt pathway boosted the Nrf2/HO-1 pathway, which augments the tissue antioxidant system, resulting in the protection of neurons [46]. In the current study, (-) pseudosemiglabrin increased Bcl-2 level, PI3K, and Akt mRNA expression while decreasing Bax, caspase-3, and the Bax/Bcl-2 ratio in a mouse model of pilocarpine-induced epilepsy. Moreover, (-) pseudosemiglabrin, in the current study, surpassed diazepam in terms of anti-apoptotic effectiveness, dramatically suppressing Bax and caspase-3, and increasing Bcl-2 production, whereas diazepam had a non-significant impact. These findings showed that (-) pseudosemiglabrin has strong anti-apoptotic and protective effects against oxidative damage caused by pilocarpine injection through modulating PI3K/Akt signaling.

The critical factor driving degenerative changes to neural tissue following seizures is the inflammatory response of brain tissue caused by seizures [48]. TLRs are pattern-recognition receptors that play a crucial role in activating the body’s innate and adaptive immunological pathways. TLR-4 is implicated in the human innate immune inflammatory process [11,49]. It is primarily expressed in microglia and astrocytes in the neurological system [8,49]. TLR-4 was shown to be involved in epileptic processes because its expression tended to trend in the opposite direction of GABARα1. Furthermore, activating GABARα1 suppressed TLR-4 and prevented epilepsy development. Additionally, TLR-4 activation suppresses GABA receptor activation at postsynaptic sites [15,49,50]. After TLR-4 activation, NF-κB, IL-1β, and TNF-α are upregulated, which enhances the activation of astrocytes and microglia that release inflammatory cytokines, increases the severity of seizures, reduces the seizure threshold, and increases the frequency of recurrence [15,16,46]. NF-κB, a transcription factor found in the TLR-4 signaling pathway, initiates and regulates the expression of several inflammatory mediators, which have a crucial role in boosting pro-inflammatory cytokine expression, including IL-1β and TNF-α [16,48]. IL-1β directly enhances neuronal excitability by blocking Ca^+2^ channel signals and lowering GABA receptor responsiveness [8,11]. Moreover, TNF-α promotes inhibitory transmission in hippocampal neurons by causing a fast and long-lasting reduction in inhibitory neuronal activity and downregulating GABA receptors [8,46].

In the current study, (-) pseudosemiglabrin effectively suppressed TLR-4, NF-κB, IL-1β, and TNF-α mRNA expressions, which were induced by pilocarpine injection, implying that it has the potential to prevent the development of epilepsy by suppressing astrocyte activation, inflammatory cytokine production, and their upstream transcription factors, TLR-4 and NF-κB, and was involved in neuroinflammation control. As a result, it improved the brain’s excitability-inhibitability balance, which explains its neuroprotective action against pilocarpine-induced neuronal damage. Similarly, in a rat inflammation model, (-) pseudosemiglabrin demonstrated substantial anti-inflammatory activity by reducing IL-1, TNF-, and NO secretion [25].

Furthermore, noncommunicable diseases, such as cardiovascular diseases, are the primary cause of mortality in the epileptic population. This is most likely due to the progressive emergence of atherosclerosis-accelerating factors, such as obesity and significant alterations in metabolic components, often known as metabolic syndrome (MS). Several studies indicate that the prevalence of MS in the epileptic population ranges from 30.6% to 52.6%. It may be connected with metabolic abnormalities caused by seizures, long-term antiepileptic medication use, a sedentary lifestyle, and other behavioral risk factors in the epileptic population. The higher risk of MS in people with epilepsy may also be attributed to a decline in quality of life, including worse mental function and the presence of psycho-emotional stress [51,52]. There is evidence that (-) pseudosemiglabrin possesses potent antidyslipidemic properties [53]. In addition, Tephrosia purpurea, which includes (-) pseudosemiglabrin, as a major ingredient, possesses potent antihyperglycemic and antihyperlipidemic characteristics [54]. Therefore, pseudosemiglabrin may be beneficial for avoiding and treating MS linked to epilepsy.

In human epilepsy and animal models of epilepsy, the hippocampus is regarded as the principal seizure starting zone, as evidenced by partial removal of the hippocampus, including the neuronally damaged area, resulting in seizure-free patients [16,38,39]. Furthermore, neuronal death is a pathophysiologic outcome of epilepsy and plays a role in epileptogenesis due to an imbalance between excitation and inhibition, oxidative stress, and neuroinflammation [11,16,29,35,38]. The current study found that pilocarpine caused hippocampal neuronal damage and loss, as evidenced by increased nuclear pyknosis, cytoplasmic vacuolation, neuronal necrosis, apoptosis, and decreased neuron viability, as well as cerebral congestion and inflammatory influx throughout various brain areas; additionally, Nissl staining of the hippocampal CA1 and CA3 regions revealed significant neurodegeneration. In contrast, (-) pseudosemiglabrin dramatically reduced pilocarpine-induced neuronal death and increased neuron survival in a mouse model of pilocarpine-induced SE. These effects may be partly mediated by (-) pseudosemiglabrin’s modulation of GABAergic and glutamatergic neurotransmission and its antioxidant, anti-apoptotic, and anti-inflammatory capabilities.

## 4. Materials and Methods

### 4.1. Chemicals and Reagents

The following chemicals and reagents were bought commercially from Sigma, St. Louis, MO, USA, pilocarpine, methyl scopolamine, phosphate-buffered saline (PBS), diazepam, dimethyl sulfoxide (DMSO), I-oxoglutarate, pyridoxal phosphate, methanol, trichloroacetic acid, ferric chloride (FeCl_3_), acetone, glutamic acid, formalin-buffered saline, hematoxylin, and eosin stains. However, Nissl’s stain was bought from VitroVivoBiotech, Gaithersburg, MD, USA, Tris-HCl buffer was supplied from Research Organics, Cleveland, OH, USA, and nuclear lysis buffer was purchased from ThermoFisher Scientific, Waltham, MA, USA.

### 4.2. Plant Materials, Extraction, and Isolation

(-) Pseudosemiglabrin, used in the present study, was extracted and isolated from *Tephrosia purpurea* L. (Pers.) as previously described [55]. Plant materials were air-dried in the shade at a controlled temperature and powdered to yield 790 g of dry powder. The extraction was performed using 95% ethanol at room temperature until exhaustion. The solvent was distilled under reduced pressure using a rotary vacuum evaporator at 40 °C to provide 53 g of residue. Part of the dried extract was reconstituted in 800 mL of 40% aqueous ethanol and fractionated using the liquid-liquid fractionation technique with light petroleum (500 mL × 3) to yield 13.45 g of petroleum ether soluble fraction, chloroform (500 mL × 4) to yield 14.12 g of chloroform soluble fraction, ethyl acetate (400 mL × 2) to yield 23.30 g of ethyl acetate soluble fraction, and the left aqueous layer was freeze-dried to yield 0.4 gm. Crystallization of the chloroform-soluble fraction afforded 250 mg of (-) pseudosemiglabrin.

### 4.3. Animals

Eight-week-old BALB/c mice weighing 20–25 g were used in the present study. Mice were obtained from the Animal Care Unit, Prince Sattam bin Abdulaziz University, Faculty of Pharmacy, Saudi Arabia, and housed in ventilated polypropylene cages at a temperature of 22 ± 2 °C with a 12 h light/dark cycle, with *ad libitum* access to water and a standard laboratory diet. Mice were acclimated for seven days before the experimental procedure. All experiments were approved by the Research Ethics Committee on the Care and Use of Laboratory Animals at Prince Sattam bin Abdulaziz University, Al-Kharj, Saudi Arabia (Registration Reference No. SCBR-056-2022), which conforms to ARRIVE guidelines and National Institutes of Health guidelines for the care and use of laboratory animals. Every effort was made to minimize animal suffering, and all animal handling occurred between 8 a.m. and 8 p.m.

### 4.4. Establishment of the Epilepsy Model in Mice

The mouse model of epilepsy was induced by intraperitoneal (i.p.) injection of 360 mg/kg pilocarpine in PBS 30 min after the i.p. injection of 1 mg/kg of methyl scopolamine to reduce the peripheral cholinergic effects of pilocarpine [9]. The negative control group was injected with PBS instead of pilocarpine. Immediately after the pilocarpine injection, mice were observed continuously for 60 min for locomotor activity and seizures. Seizures were scored according to the Racine scale, where seizure severity was graded as follows: 0 = unresponsive; 1 = hyperactivity and vibrissae twitching; 2 = head nod, head clonus, and myoclonic jerks; 3 = unilateral forelimb clonus; 4 = rearing with bilateral forelimb clonus; and 5 = generalized tonic-clonic seizure with loss of righting reflex. The latency to the first seizure and the percentage of SE were also recorded, with the convulsion described as the occurrence of Racine grade 4–5 seizures and the SE described as continuous seizures lasting at least 5 min, or seizures that have recurred at short intervals (less than a minute). When mice suffered grade 4–5 or SE seizures for 60 min, the seizures were relieved by an i.p. injection of 1 mg/kg diazepam to reduce mortality [8].

### 4.5. Experimental Protocol

In the present study, 110 mice were used. They were randomly and blindly allocated into six groups. Group I (CON, n = 10 mice), normal mice were administered PBS intraperitoneally (i.p.) in a volume equivalent to pilocarpine. Group II (PIL, n = 20 mice) was convulsion-induced mice by an i.p. injection of 360 mg/kg pilocarpine [9]. Group III (DIZ, n = 20 mice) was the positive control group of convulsion-induced mice, orally treated with a single dose of 5 mg/kg diazepam 1 h before pilocarpine injection [34]. Group IV, V, and VII (SSL, SSM, and SSH, n/group = 20 mice) were convulsion-induced mice, orally treated with a single dose of 12.5, 25, and 50 mg/kg (-) pseudosemiglabrin dissolved in 0.5% DMSO, respectively, 1 h before pilocarpine injection [25]. All oral treatments were administered intragastrically with the feeding syringe. Seventy-two hours after the pilocarpine injection, the mice were euthanized by cervical dislocation, and their brains were harvested and weighed wet. The left hemisphere was preserved in 10% formalin-buffered saline for further histopathological evaluation. However, the right hemisphere’s cortex and hippocampus were dissected, then divided into two halves; the first half was homogenized in ice-cold PBS (pH = 7.4) to obtain a 10% *w*/*v* homogenate, centrifuged at 5000 rpm at 4 °C for 10 min, and the supernatant collected. However, the pellet was resuspended in ice-cold nuclear lysis buffer, the suspension was centrifuged at 5000 rpm at 4 °C for 10 min, and the supernatant was collected. The remaining half was homogenized in ice-cold lysis buffer to obtain a 10% *w*/*v* homogenate. The brain tissue homogenate and supernatants were stored at −80 °C and used to evaluate the brain tissue biochemical, nuclear, and quantitative real-time polymerase chain reaction (qRT-PCR) parameters.

### 4.6. Assessment of Mice Locomotor Activities

Immediately after pilocarpine injection (60 min after oral treatment with (-) pseudosemiglabrin and diazepam), mice’s locomotor activities were evaluated to assess the sedative effect of (-) pseudosemiglabrin using the methods described by Kandeda et al., (2022). The open maze consists of a wooden box with a length of 20 cm, a width of 20 cm, and a height of 45 cm. The exploration area was divided into 16 squares (5 cm × 5 cm). Mice were placed in the center of the experimental device. The behavior of each mouse was monitored for 5 min. In addition, several behavioral parameters were evaluated, including the number of crossings (total quadrants overpassed), rearings (times the mouse stood on its hind legs), groomings (mouse self-cleaning behavior), and immobility, as well as latency to initiate locomotion [35].

### 4.7. Assessment of Total Protein Content

The total protein content of brain tissue was determined according to the manufacturer’s instructions using a colorimetric assay kit available from AssayGenei, Dublin, Ireland (Cat. # BA0168), with a minimum detection limit of 0.06 mg/mL. Samples absorbances were analyzed using a Stat Fax 2100 automated plate reader, Fisher Bioblock Scientific, BP, Illkirch Cedex, France, and results were expressed as mg protein/mL brain tissue homogenate.

### 4.8. Assessment of Brain GABA and Glutamate Levels

Brain GABA and glutamate levels were determined according to the manufacturer’s instructions using colorimetric assay kits available from Antibodies-Online GmbH, Limerick, PA, USA (Cat. # ABIN6966944) and LifeSpan BioSciences, Inc., Seattle, WA, USA (Cat. # LS-F55498), with minimum detection limits of 18.75 pg/mL or 48.2 ng/mL. The absorbances of the samples were analyzed on a Stat Fax 2100 automated plate reader, and the results were expressed as ng/g brain tissue.

### 4.9. Assessment of the GABA Transaminase and Glutamate Decarboxylase Activities

GABA transaminase and glutamate decarboxylase activities of brain tissue were analyzed colorimetrically as defined by Kandeda et al., (2021) and Kandeda et al., (2022) respectively. Briefly, GABA transaminase activity was assessed by adding 15 mM I-oxoglutarate, 15 mM GABA, 10 mg pyridoxal phosphate, and 0.1 mL brain tissue homogenate supernatant to 10 mL tubes, and the volume was made up to 3 mL by addition of Tris-HCl buffer (50 mM, pH 7.4). The mixture was then incubated for 30 min at 37 °C. The blank was prepared by adding 0.1 mL of 5% methanol instead of the homogenate supernatant. The reaction was stopped by adding 0.5 mL of 20% trichloroacetic acid. Then, the color complex of succinic semialdehyde and 3-methyl-2-benzothiazole-2-hydrazone was developed in the presence of 1 mL of 12% FeCl_3_ (pH 2), followed by 5 min addition of 4 mL of acetone and evaluated after 30 and 90 s at 610 nm using a Biosystems semi-automated analyzer (BTS-350, Barcelona, Spain) against the blank. Brain tissue GABA transaminase activity was expressed in units/g brain tissue, where one unit corresponds to the production of 1 nM succinic semialdehyde/g wet tissue/min [31]. However, glutamate decarboxylase activity in brain tissue was measured by adding 200 μL of brain tissue homogenate supernatant to 200 μL of 0.05 M glutamic acid (neutralized to pH 6.7) and then 20 μL of pyridoxal-5-phosphate (50 mM) and incubating the combination for 30 min at 37 °C. The reaction was stopped by heating the mixture to 60 °C for 10 min. In place of the brain tissue homogenate supernatant, 200 µL of Tris-based buffer was added to the mixture in blank samples. The increase in GABA was analyzed after 30 and 90 s at 455 nm using a Biosystems semi-automated analyzer against the blank. One unit of enzyme activity corresponds to the formation of 1 μmol of GABA/g of wet tissue/min [35].

### 4.10. Assessment of Brain GRIN1, GABARα1, SLC6A1 and SLC1A2

The brain GRIN1, GABARα1, SLC6A1, and SLC1A2 were evaluated by measuring their brain protein levels and mRNA expressions. The brain GRIN1, GABARα1, SLC6A1, and SLC1A2 protein levels were assessed using the manufacturer’s instructions of ELISA kits bought from MyBioSource Inc., San Deigo, CA, USA, Abbexa LLC., Sugar Land, TX, USA, and Antibodies-Online GmbH, Aachen, Germany (Cat. # MBS4503534, MBS9339158, abx544919, and ABIN6959549), respectively, with minimum detection limits of 0.122 ng/mL for the GRIN1 kit, 0.1 μmol/L for the GABARα1 kit, and 0.054 ng/mL for the SLC6A1 and SLC1A2 kits. A Stat Fax 2100 automated plate reader was used to detect brain GRIN1, SLC6A1, SLC1A2, and GABARα1 levels, and expressed as pg and nmol/mg tissue protein.

### 4.11. Assessment of Brain Tissue Oxidative Stress Indicators

Oxidative stress indicators were assessed by determining brain tissue MDA, GSH, NO levels, SOD activity, HO-1, and Nrf-2 mRNA expression. MDA, GSH, NO levels, and SOD activity in brain tissue were assessed using the manufacturer’s instructions of colorimetric assay kits bought from MyBioSource Inc., San Deigo, CA, USA (Cat. # MBS2540407, MBS2540412, MBS2540418, and MBS2540402). Brain tissue MDA, GSH, and NO levels were expressed as nmol/mg tissue protein, and SOD activity was expressed as U/mg tissue protein. Brain tissue oxidative stress markers’ absorbances were analyzed using a Stat Fax 2100 automated plate reader.

### 4.12. Assessment of Apoptosis Markers

Brain tissue apoptosis was assessed by measuring caspase-3, Bax, and Bcl-2 protein levels, the mRNA expression of PI3K, and Akt. Caspase-3, Bax, and Bcl-2 protein levels were measured using the manufacturer’s instructions of sELISA kits purchased from MyBioSource (Cat. # MBS265309, MBS2607437, and MBS263525, respectively). Brain tissue apoptosis markers’ levels were analyzed by an automated ELISA plate reader, Stat Fax 2100, and were expressed as pg and ng/mg tissue protein, and Bax/Bcl-2 ratio.

### 4.13. Assessment of Inflammatory Signals

Brain tissue inflammatory signals were assessed by evaluating brain tissue TLR-4, NF-κB, IL-1β, and TNF-α mRNA expressions.

### 4.14. Evaluation of mRNA Expressions

The quantitative real-time polymerase chain reaction was used to quantify the mRNA expressions of brain GRIN1, GABARα1, SLC6A1, SLC1A2, HO-1, Nrf2, PI3K, AKT, NF-κB, IL-1β, TNF-α, and TLR-4. The total RNA was extracted from brain tissues utilizing a total RNA extraction kit (RNAsimple Total RNA Kit, Tiangen Biotech, Beijing, China, Cat. # 4992858) according to the manufacturer’s instructions. The iScript cDNA Synthesis Kit was used for reverse transcription (Bio-Rad, Hercules, CA, USA, Cat. # 1708891). The cDNA obtained was amplified with a real-time PCR system (qTOWER, Analytik Jena GmbH, Jena, Germany), with the thermal cycling conditions as follows, the initial denaturation and enzyme activation at 95 °C for 3 min (1 cycle), and denaturation at 95 °C for 15 s (sec), annealing at 55 °C for 30 s, and extension at 72 °C for 30 s (40 cycles) using iQ SYBR Green Supermix (Bio-Rad, Hercules, CA, USA, Cat. # 1708880). The melting curve was performed after the cycle to ensure no non-specific products were present (1 cycle). Relative mRNA expression was normalized to β-actin or GADPH as an internal control to standardize the difference, and the primers’ sequences are shown in Table 3.

### 4.15. Histopathological Evaluation

Brain tissues were processed as described by Suvarna et al. (2018). Briefly, left hemispheres were fixed in 10% formalin-buffered saline, embedded in paraffin, cut into 5 μm sections, and stained with H and E and Nissl’s stain for further evaluation of the histopathological changes [56]. Sections were analyzed by a blinded pathologist using a light microscope (Leica Universal Microscope, Wetzlar, Germany). Histopathological changes in ten randomly selected, non-overlapping H and E sections per slide were arbitrarily scored for the presence of cerebral congestion, nuclear pyknosis, cytoplasmic vacuolation, neuronal necrosis, and inflammatory influx, using a 0–3 scale, where 0 is no histological change, 1 is mild, 2 is moderate, and 3 is severe changes influencing brain tissue sections. Moreover, ten randomly selected, non-overlapping Nissl’s-stained sections per slide were analyzed to count the number of surviving hippocampal CA1 and CA3 pyramidal cells.

### 4.16. Statistical Analysis

The present study’s data were assessed using the Statistical Package for the Social Sciences Statistics Software for Windows (IBM-SPSS, version 25, Armonk, NY, USA), and the data were expressed as mean ± SD. The groups’ data were compared with a one-way analysis of variance (ANOVA) followed by Dunnett’s T3 multiple comparison post-hoc test to determine significant differences between individual groups. A *p*-value < 0.05 is considered significant.

## 5. Conclusions

Data from the current study revealed that (-) pseudosemiglabrin therapy successfully alleviates pilocarpine-induced epilepsy. This potentiality may be partly mediated by enhancing GABAergic neurotransmission while decreasing glutamatergic neurotransmission and its antioxidant, anti-apoptotic, anti-inflammatory, and neuroprotective properties. Furthermore, (-) pseudosemiglabrin displayed anti-epileptic efficacy comparable to diazepam, the standard anti-epileptic medication, in this animal model without sedation induction. As a result, (-) pseudosemiglabrin can be a feasible and safer medication for the management of epilepsy.

## Figures and Tables

**Table 1:** (-) Pseudosemiglabrin mitigated pilocarpine-induced convulsions and mortality. Results were expressed as mean ± SD and percentage.

Group	Latency to First Convulsion (s)	Convulsion (%)	SE (%)	Seizures Severity Score	Survival (%)
**CON**	-	0%	0%	-	100%
**PIL**	370.7 ± 46.8	100%	100%	4.70 ± 0.47	30%
**DIZ**	-	0%	0%	-	95%
**SSL**	484.3 ± 65.2	70%	50%	3.85 ± 0.67 *	50%
**SSM**	1180.5 ± 319.4 ***	30%	10%	1.85 ± 1.60 ***	80%
**SSH**	-	0%	0%	-	100%

CON: control group; PIL: pilocarpine-induced convulsion group; DIZ: convulsion-induced group pretreated by single oral dose of diazepam 5 mg/kg; SSL: convulsion-induced group pretreated by single oral dose of (-) pseudosemiglabrin 12.5 mg/kg; SSM: convulsion-induced group pretreated by single oral dose of (-) pseudosemiglabrin 25 mg/kg; SSH: convulsion-induced group pretreated by single oral dose of (-) pseudosemiglabrin 50 mg/kg. * *p* < 0.05 and *** *p* < 0.001 (vs. PIL group).

**Table 2:** (-) Pseudosemiglabrin enhanced the suppressed mice’s locomotor activities induced by pilocarpine injection. The result was expressed as mean ± SD.

Group	Number of Crossings	Number of Rearings	Number of Groomings	Number of Immobility	Latency to Initiate Locomotion (s)
**CON**	44.6 ± 4.88 ***	18.1 ± 2.08 ***	25.8 ± 1.69 ***	3.1 ± 0.57 ***	1.95 ± 0.21 ***
**PIL**	26.9 ± 5.19	10.45 ± 1.77	12.15 ± 3.39	14.2 ± 2.09	11.24 ± 2.19
**DIZ**	9.45 ± 1.85 ***^+++^	7.1 ± 1.37 ***^+++^	6.05 ± 1.28 ***^+++^	18.9 ± 4.52 ***^+++^	15.7 ± 3.23 ***^+++^
**SSL**	27.25 ± 4.52 ^+++^	11.15 ± 2.46 ^+++^	14.9 ± 2.53 *^+++^	10.7 ± 2.01 ***^+++^	8.86 ± 2.29 **^+++^
**SSM**	31.55 ± 5.22 *^+++^	12.2 ± 3.01 ^+++^	17.5 ± 2.57 ***^+++^	7.1 ± 1.25 ***	4.66 ± 1.06 ***
**SSH**	40.65 ± 5.7 ***	15.6 ± 2.44 ***	22 ± 2.88 ***	5.15 ± 0.99 ***	2.96 ± 0.60 ***

CON: control group; PIL: pilocarpine-induced convulsion group; DIZ: convulsion-induced group pretreated by single oral dose of diazepam 5 mg/kg; SSL: convulsion-induced group pretreated by single oral dose of (-) pseudosemiglabrin 12.5 mg/kg; SSM: convulsion-induced group pretreated by single oral dose of (-) pseudosemiglabrin 25 mg/kg; SSH: convulsion-induced group pretreated by single oral dose of (-) pseudosemiglabrin 50 mg/kg. * *p* < 0.05, ** *p* < 0.01 and *** *p* < 0.001 (vs. PIL group) and ^+++^ *p* < 0.001 (vs. SSH group).

**Table 3:** Primers used for quantitative real-time PCR.

mRNA	Forward	Reverse
**GRIN1**	CGATGACCACGAGGGCCGGG	GGCATTCCCAGAGATCTCGCG
**GABARα1**	TGAGCACACTGTCGGGAAGA	CAGCAGTCGGTCCAAAATTCT
**SLC6A1**	GGTGTTGGTTGGACTGGAAAGGTG	AAGCGTCACTCCACGGAAGAAC
**SLC1A2**	GGTCATCTTGGATGGAGGTC	ATACTGGCTGCACCAATGC
**HO-1**	GATAGAGCGCAACAAGCAGAA	CAGTGAGGCCCATACC AGAAG
**Nrf2**	TCTTGGAGTAAGTCGAGAAGTGT	GTTGAAACTGAGCGAAAAAGGC
**TLR-4**	ATGGCATGGCTTACACCACC	GAGGCCAATTTTGTCTCCACA
**NF-κB**	ACGACATTGAGGTTCGGTTC	ATCTTGTGATAGGGCGGTGT
**IL-1β,**	CACTACAGGCTCCGAGATGA	TTTGTCGTTGCTTGGTTCTC
**TNF-α**	GGCAGGTCTACTTTGGAGTCATTGC	ACATTCGAGGCTCCAGTGAATTCGG
**AKT**	CATGAGGATCAGCTCGAACAGC	ACGGGCACATCAAGATAACGG
**PI3K**	TTCCCTCGCAATAGGTTCTCC	GACCAATACTTGATGTGGCTGAC
**β-actin**	ACCGTGAAAAGATGACCCAGA	ATGGGCACAGTGTGGGTGA
**GADPH**	GGAGCGAGATCCCTCCAAAAT	GGCTGTTGTCATACTTCTCATGG

## Data Availability

The data presented in this study are available on logic request from the corresponding author.
